# Redesigning Surveillance through the Global Emerging Infections Surveillance Program

**DOI:** 10.3201/eid3014.240832

**Published:** 2024-11

**Authors:** David M. Brett-Major

**Affiliations:** College of Public Health, University of Nebraska Medical Center, Omaha, Nebraska, USA

**Keywords:** Antimicrobial resistance, vector-borne infections, viruses, zoonoses, bioterrorism and preparedness, surveillance, parasites, sexually transmitted infections, malaria, Global Emerging Infections Program, Department of Defense, United States

This supplement contains new technical outputs and perspectives from the Global Emerging Infections Surveillance (GEIS) program within the Department of Defense (DoD). GEIS develops, invests in, disseminates, and integrates information from health surveillance activities conducted by military, academic, public health, and other partners. GEIS-funded researchers from around the world provide novel surveillance data that are used to update the military and civilian outputs from this long-standing initiative. 

In this journal supplement, J. Early et al. provide an overview of the GEIS program’s history and mission (*1*). Readers outside of the military health system might be unfamiliar with GEIS programming and outputs that have broader applications for public health considerations. The aspects of how this work occurred might also come as a surprise.

DoD emerging infectious disease investigators comprise a diverse group, and their collaborators are equally diverse. US taxpayers have been investing in networked surveillance and science through the DoD research and development enterprise for decades. That investment has kept pace with technological norms, including advanced sequencing techniques and bioinformatics. Work funded directly and indirectly by GEIS takes place in nearly every subregion of the globe and intersects with many normative US and international surveillance initiatives. Although GEIS often focuses on health protection needs of globally deployed military personnel, many nonmilitary stakeholders also benefit from its technical outputs. Such beneficiaries include those who are concerned for pandemic threats like SARS-CoV-2, threats posed to less well studied at-risk populations (e.g., rural persons exposed to ticks on the Eurasian steppes [*2*]), and the characterization of threats for which risks are not yet understood (e.g., bandavirus in Thailand [*3*]).

Three implications from this work are relevant to next steps in surveillance design and investment for the GEIS program and others who are not embedded in that enterprise. First, multidisciplinary efforts enable more contextualized findings that can be applied to use-case–based risk assessments and program improvements. Consider, for instance, the contrasting contexts in this supplement of the outbreak investigations into scrub typhus from trombiculid mites among military personnel in Australia (*4*) and the metagenomic sequencing of tickborne pathogens in Mongolia (*2*). In Australia, we see the end of a risk lifecycle and its human consequences; in Georgia, we see the beginning of risk and the enzootic findings of possible exposures not yet realized. Renewed interest in team science and multidisciplinary practice may enable funding and implementation approaches that integrate such assessments, in turn enabling more reliable interactions between clinical and public health actors, as well as entomologic, zoologic, social science, and other researchers.

Second, cross-cutting themes that matter in any setting matter in each setting. Several articles in the supplement address antimicrobial resistance (AMR) (*5*–*8*). Whether in travelers’ diarrhea, infectious consequences of trauma, sexually transmitted infections, malaria, or its many other contexts, AMR forces incorporation of analytic approaches of interpathogen and interhost differences and environmental influences. Sponsors like GEIS and their implementers have an opportunity to advance pathogen-agnostic surveillance approaches, which can be done in part through continued innovation in AMR risk management and other broad challenges in ways that are applicable across a variety of pathogens.

Third, novel technologies require validation based on the intended application (*9*). For instance, in the realm of genomic sequencing, the capability to generate a nucleic acid sequence is increasingly common, as is the ability to align and upload sequences to shared databases. Add to those capabilities other new online opportunities for structurally sound proteomic translation and we can quickly appreciate the vast data-verse enabled by such information. However, differences between hosts, host populations, vectors, and environments make over-extrapolation of any single sequencing result problematic. Assessing sequencing results to determine whether a primer for a nucleic acid test applies to a pathogen variant is entirely different than assessing sequencing results to examine mechanisms that cause immune escape from existing vaccines. In addition to progress in data integration and advanced analytics, use-case anchored question design also helps us navigate the complexities arising from examining diverse data sources and types.

Thank you for your interest this supplement. And many thanks to the programming and implementation efforts of the authors.
